# Genotype-independent *Agrobacterium rhizogenes*-mediated root transformation of chickpea: a rapid and efficient method for reverse genetics studies

**DOI:** 10.1186/s13007-018-0315-6

**Published:** 2018-07-06

**Authors:** Pooja Rani Aggarwal, Papri Nag, Pooja Choudhary, Niranjan Chakraborty, Subhra Chakraborty

**Affiliations:** National Institute of Plant Genome Research, Aruna Asaf Ali Marg, New Delhi, 110067 India

**Keywords:** Legumes, *Cicer arietinum*, *Agrobacterium rhizogenes*, strain K599, Transformation efficiency, Functional genomics, Green fluorescent protein (GFP) expression, TRANSPARENT TESTA 2, Proanthocyanidins, Fungal infection

## Abstract

**Background:**

Chickpea (*Cicer arietinum* L.), an important legume crop is one of the major source of dietary protein. Developing an efficient and reproducible transformation method is imperative to expedite functional genomics studies in this crop. Here, we present an optimized and detailed procedure for *Agrobacterium rhizogenes*-mediated root transformation of chickpea.

**Results:**

Transformation positive roots were obtained on selection medium after two weeks of *A. rhizogenes* inoculation. Expression of green fluorescent protein further confirmed the success of transformation. We demonstrate that our method adequately transforms chickpea roots at early developmental stage with high efficiency. In addition, root transformation was found to be genotype-independent and the efficacy of our protocol was highest in two (Annigiri and JG-62) of the seven tested chickpea genotypes. Next, we present the functional analysis of chickpea hairy roots by expressing *Arabidopsis TRANSPARENT TESTA 2* (*AtTT2*) gene involved in proanthocyanidins biosynthesis. Overexpression of *AtTT2* enhanced the level of proanthocyanidins in hairy roots that led to the decreased colonization of fungal pathogen, *Fusarium oxysporum*. Furthermore, the induction of transgenic roots does not affect functional studies involving infection of roots by fungal pathogen.

**Conclusions:**

Transgenic roots expressing genes of interest will be useful in downstream functional characterization using reverse genetics studies. It requires 1 day to perform the root transformation protocol described in this study and the roots expressing transgene can be maintained for 3–4 weeks, providing sufficient time for further functional studies. Overall, the current methodology will greatly facilitate the functional genomics analyses of candidate genes in root-rhizosphere interaction in this recalcitrant but economically important legume crop.

**Electronic supplementary material:**

The online version of this article (10.1186/s13007-018-0315-6) contains supplementary material, which is available to authorized users.

## Background

The family Leguminosae is comprised of economically important legume crops, which are widely grown for grain and forage purposes [1]. The United Nations has declared 2016 as the ‘International Year of Pulses’, affirming the need to focus on the role that legumes can play in ensuring food security [2]. Chickpea (*Cicer arietinum* L.) is the second most widely grown pulse crop and serves as an important source of dietary protein [3]. It is cultivated for food and fodder in the semi-arid environment and poorly fertilized soil. Although chickpea is grown in more than 40 countries; South and Southeastern Asia are main growing regions, where India is the major contributor with approximately 67% of global annual production [4]. However, there has been stagnancy in chickpea production due to various biotic and abiotic stress factors [5, 6].

Similar to other legumes chickpea has a narrow genetic base which impacts its use in genetics and breeding for crop improvement. Despite the nutritional and commercial importance of chickpea, less is known about the pathways and genes responsible for agronomic traits because of its recalcitrant nature [7]. Recent efforts to sequence the expressed sequence tags (ESTs) [8, 9], transcriptome [10–12] and genome [3, 13] along with high-throughput proteome analyses [14–16] have led to the identification of novel genes, transcripts and proteins involved in several regulatory processes. However, tools for gene function analysis are very limited in chickpea. Determining the function of genes/proteins identified through various such large scale OMICS studies is a major challenge, especially in this recalcitrant crop for conventional transformation methods. An easy, reproducible and efficient method of plant transformation protocol is thus crucial for functional studies and crop improvement program. Introduction of new gene or modulation of the expression of an endogenous gene in a native system causes phenotypic variation that can be employed further for the elucidation of gene function. So far, few efforts have been made with standard *Agrobacterium tumefaciens*-mediated transformation for developing transgenic chickpea [17–21]. However, the transformation efficiency achieved in these studies was very low, genotype dependent and the approach is laborious as well as time consuming. To circumvent these shortcomings, a rapid, efficient and genotype-independent transformation method is a pre-requisite for functional genomics studies in this important grain legume.

Root transformation using *A. rhizogenes* has emerged as an alternative to traditional transformation and breeding strategies that is gaining importance as an important tool for reverse genetics studies in plants, especially legumes [22]. Limpens et al. have shown that RNA interference in *A. rhizogenes* transformed roots also serve as a valuable approach in *Arabidopsis* and *Medicago* [23]. It is an efficient method of choice for quick over-expression or knock-out of genes in roots. The regenerated roots are also expected to be nonchimeric as they originate from single cells [24]. Recently, with the generation of disarmed *A. rhizogenes, rol* genes have been removed from the Ri plasmid which reduces the extent of undesirable hair-like root formation [25]. Owing to these advantages, regeneration of transgenic plantlets using *A. rhizogenes* has also been reported in several plants, including *Nicotiana* spp. [26], *Ipomoea batatus* [27], *Brassica oleracea*, *Brassica campestris* [28] and *Spinacia oleracea* [29].

Hairy root cultures have simplified the elucidation of root development and organ-specific response during root-pathogen and root-rhizosphere interactions. A key milestone among the wide applications of *A. rhizogenes*-mediated root transformation is the generation of composite plant consisting of transgenic root and wild-type shoot [30–32]. Composite plants serve as an ideal system for (1) studying root biology, (2) gene function studies in association with other organisms and (3) root-pathogen interaction studies. For example, pathogenicity of the soil-borne fungal pathogen *Fusarium solani* and the impact of isoflavanoid accumulation on pathogen invasion after hairy root development were studied in susceptible as well as partially resistant cultivars of soybean [33]. Similarly, propagation of cyst nematode in soybean [34, 35], infection with mycorrhizal fungi in bindweed [36] and infection processes of obligate fungal parasites in strawberry [37] were studied by developing *A. rhizogenes*-mediated hairy roots. In addition, hairy root generation has been employed to study root and nodule development in alfalfa [38, 39], common bean [40], pea [41]; rhizobial colonization and nitrogen fixation in lotus [42, 43]. However, shoot evaluation using this method is only applicable where transgenic plants have been regenerated from *A. rhizogenes*-transformed hairy roots [26–29, 44]. In the past, *A. rhizogenes*-mediated root transformation has also been reported in chickpea [45, 46], however the efficiency of the method described in these studies was low with a variable degree of success (Additional file 1: Table S1).

Here, for the first time we report the method for *A. rhizogenes*-mediated highly efficient root transformation in different chickpea cultivars. Further, we demonstrate the expression of *Arabidopsis* MYB family transcription factor TRANSPARENT TESTA 2 (TT2) [47] in chickpea hairy roots that resulted in the massive accumulation of oligomeric proanthocyanidins (PAs). This method can be useful for large scale over-expression and knock-down studies of genes of interest in chickpea. In accordance, chickpea can be utilized as a model system to address important biological questions unique particularly to this legume crop and improving agronomic traits using our high-throughput root transformation method.

## Results and discussion

### Induction of hairy roots in chickpea using *Agrobacterium rhizogenes*

**Fig. 1:**
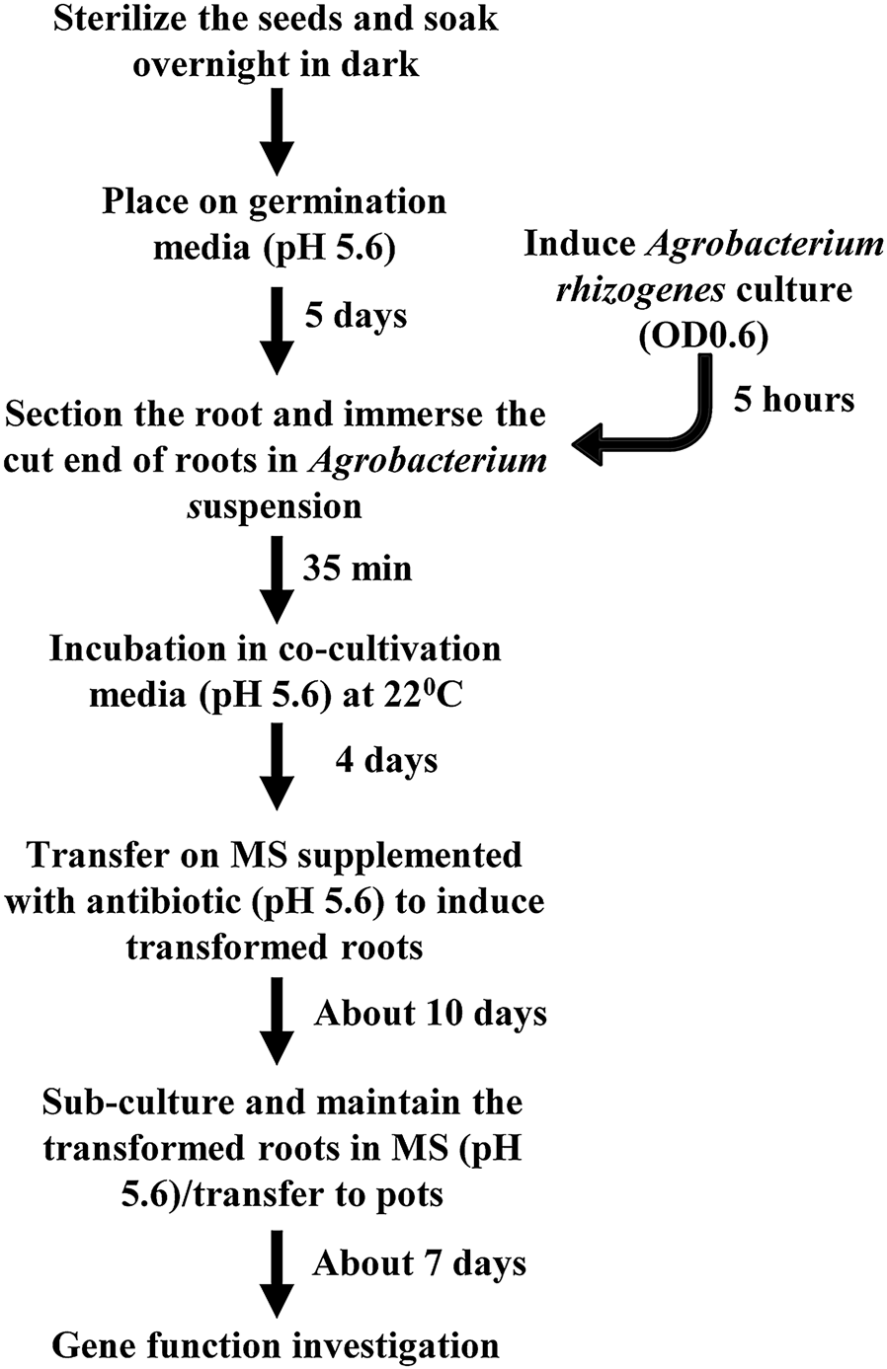
Schematic representation of the timeline required for *Agrobacterium rhizogenes*-mediated root transformation of *Cicer arietinum*

*Agrobacterium rhizogenes*-mediated root transformation in chickpea was performed following the protocol developed for *Phaseolus* with few modifications [40]. Prior to transformation, the binary vectors were transferred into competent *A. rhizogenes* strain K599 by electroporation as described earlier [48]. The suitability of K599 strain in the root transformation of legumes, including *Glycine max* [30] and *Phaseolus* spp. [40, 49] has been demonstrated previously. Transformation steps used for hairy root development are given in Fig. 1. Briefly, chickpea seeds were grown on MS salt medium (pH 5.6). Five days old seedlings were used for transformation. Preliminary experiments have revealed that transformation efficiency did not differ significantly for 5–7 days old seedlings. However, satisfactory results were not obtained beyond seventh day (data not shown). Roots were cut below epicotyl near the cotyledonary region and submerged into the suspension of *A. rhizogenes* K599 at optical density (OD_600_) of 0.6. Slanting cut was made to increase the surface area for optimum bacterial contact. Alternatively, primary roots were also inoculated using cotyledonary injection method [40] and primary screening was performed by evaluating the number of explants with antibiotic selection positive roots. The efficiency of transformation on antibiotic selection medium was higher (73.33%) using immersion method than the cotyledonary injection method (38.66%) (Table 1). Therefore, immersion method of inoculation was used for subsequent studies and validation of the transformed roots.

**Table 1:** Primary screening with different methods used for root transformation. Efficiency of two different methods used for root transformation of chickpea (*Cicer arietinum* cultivar Annigeri) seedlings

Method used for hairy root transformation by *A. rhizogenes* K599	Total number of explants inoculated	Explants with antibiotic selection positive roots (%)
Infection through cotyledonary node injection in intact seedlings^**^	50	38.66 (± 3.05)^b*^
Infection at the cut end of the hypocotyl^**^	50	73.33 (± 1.15)^a^

Each value represents the mean of three independent experiments with standard deviation (SD). Approximately 50 seedlings were examined for each individual experiment

^∗^Values in the same column followed by different letters showed significant difference among different transformation methods (Fisher’s LSD test, *P *< 0.05)

^**^Co-cultivation was done at 22 °C for 4 days

In addition, both MS salt and water were used to resuspend the *Agrobacterium* and no significant difference was found in the root transformation efficiency (Table 2). It has been shown previously that induction of bacterial suspension with acetosyringone improves the transformation efficiencies and stability [50]. Both the bacterial media listed in Table 2 were supplemented with 100 µM acetosyringone.

**Table 2:** Efficiency of root transformation of *C. arietinum* (cultivar Annigeri) with different bacterial suspension media

Bacterial suspension media	Total number of explants inoculated	Explants with antibiotic selection positive roots (%)
MS salt^**^	50	72.50 (± 2.5)^a*^
Water^**^	50	73.33 (± 3.8)^a^

Each value represents the mean of three independent experiments with standard deviation (SD). Approximately 50 seedlings were examined for each individual experiment

^∗^Values in the same column followed by same letter showed no significant difference among different suspension medium (Fisher’s LSD test, *P *< 0.05)

^**^Co-cultivation was done at 22 °C for 4 days

### Optimization of co-cultivation conditions

**Fig. 2:**
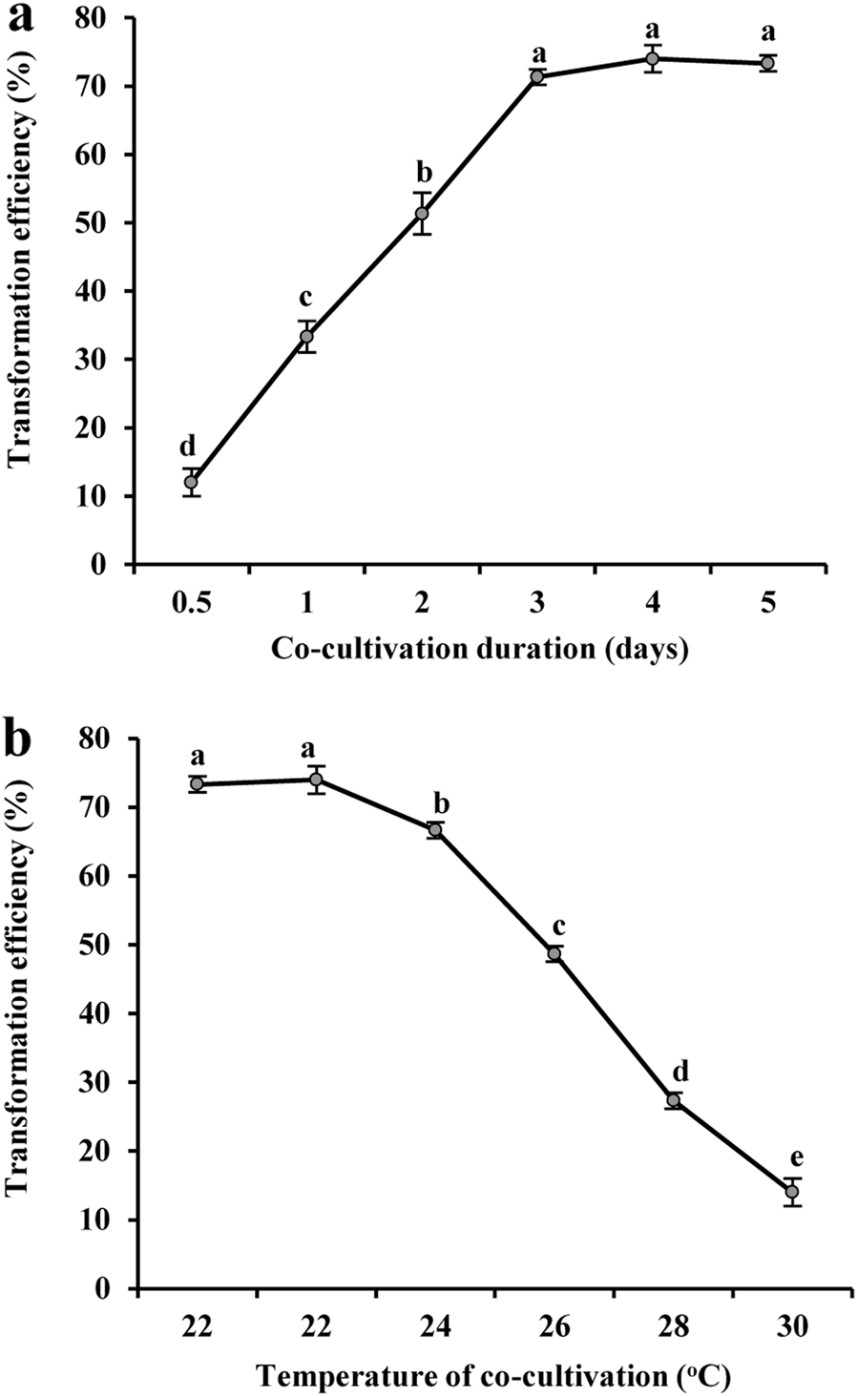
Effects of co-cultivation conditions on transformation efficiency of *C. arietinum* cultivar Annigeri. **a** Effects of duration of co-cultivation, **b** temperature during co-cultivation on transformation frequency were determined. Each value represents the mean of three independent experiments with standard deviation (SD). Approximately 50 seedlings were examined for each individual experiment. Values with different letters are significantly different at *P *< 0.05 (Fisher’s LSD test)

**Fig. 3:**
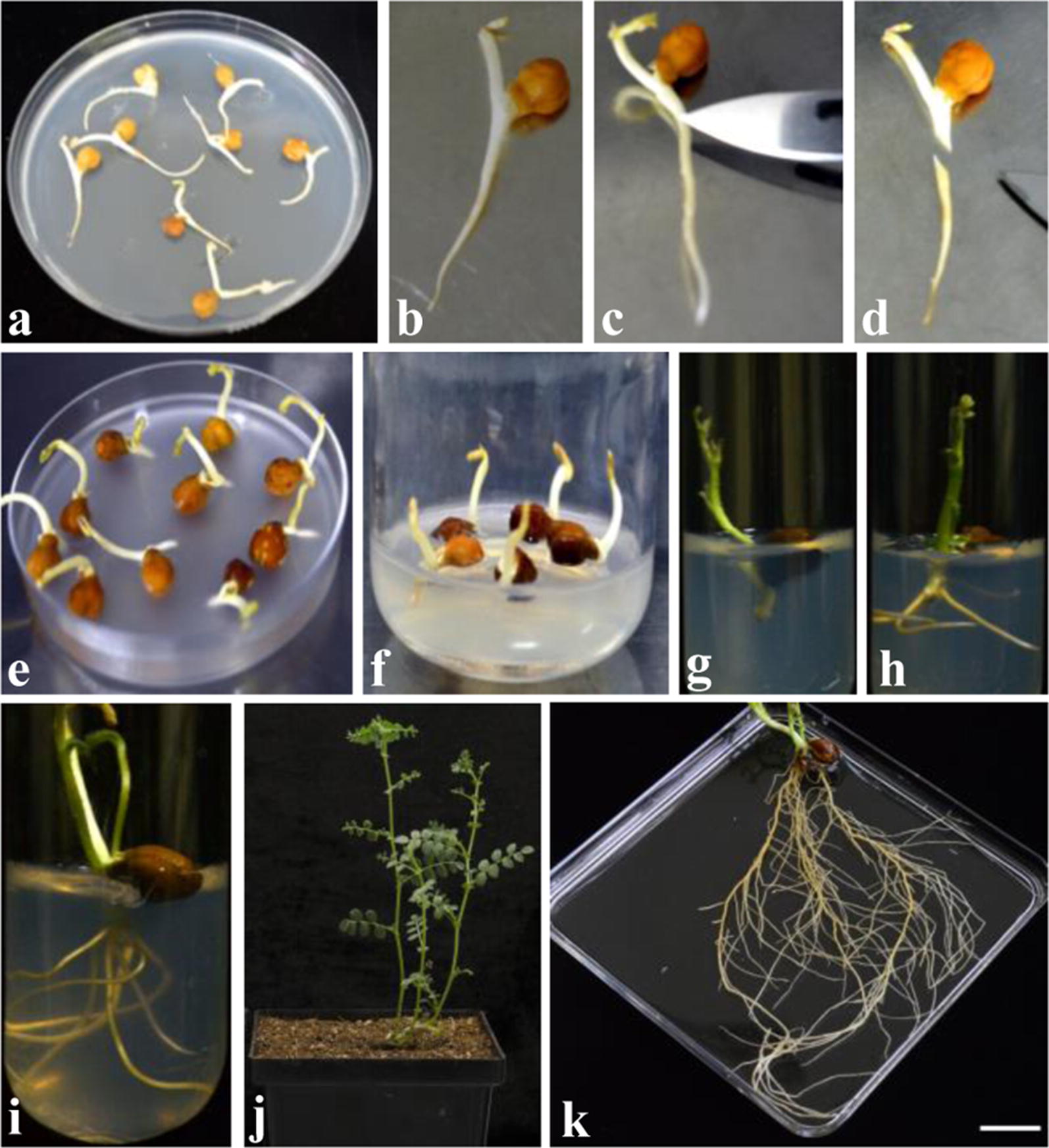
Illustrations depicting the main steps for the root transformation of chickpea cultivar Annigeri. **a** Plants grown for 5 days in MS medium, **b** germinated chickpea seedling, **c**, **d** sectioning the root near hypocotyl region, **e** immersing the cut end into *A. rhizogenes* suspension, **f** explant transferred in co-cultivation medium, **g** explant transferred to selection medium after co-cultivation, **h**, **i** transformed roots grown in the selection medium after 3 and 7 days respectively. After 7 days, plantlets were transferred to pots, **j** plants grown in pot after 15 days, **k** completely grown hairy root system in chickpea. Scale bar represents 10 mm

After inoculation, seedlings were transferred to the co-cultivation media. The marked effect of temperature and co-cultivation duration on transformation efficiency has previously been reported using both *A. rhizogenes* and *A. tumefaciens* [51–53]. We also optimized the co-cultivation temperature and duration to achieve maximum efficiency. For obtaining optimum conditions, various co-cultivation durations (0.5, 1, 2, 3, 4 and 5 days) and temperatures (20, 22, 24, 26, 28 and 30 °C) were evaluated. Interestingly, transformation frequencies were found to be increased linearly from 0.5 to 3 days of co-cultivation (12-71.33%), exhibiting maximum efficiency at 4 days (74%) (Fig. 2a). After 4 days, no significant difference was observed in the efficiency. Next, we observed a slight increase in the transformation efficiency from 20 °C (73.3%) to 22 °C (74%). Root transformation efficiencies were decreased from 74 to 14% with the increasing temperature beyond 22 °C up to 30 °C (Fig. 2b). It is interesting to note that 22 °C was reported as the optimum co-cultivation temperature in *Lotus* exhibiting 93.59% transformation frequency during *A. rhizogenes*-mediated root transformation [54]. Based on these observations, co-cultivation at 22 °C for 4 days was found to be optimum for chickpea root transformation. Subsequently, the seedlings were transferred to medium supplemented with 20 mg L^−1^ hygromycin and placed in light. Roots started emerging after 5-6 days of inoculation. The stages mentioned above are shown in Fig. 3a–i. In contrast, no roots were developed from the control plants inoculated with water on media supplemented with 20 mg L^−1^ hygromycin (Additional file 2: Fig. S1).

For comparative studies in transformed and untransformed roots, control plants treated with water were grown on medium without any antibiotic while transformed plants were grown on medium containing antibiotic. Two week post inoculation, plantlets were transferred to the pots and grown in the growth chamber (Fig. 3j). Roots grown outside an aseptic environment without selection showed increased lateral branching at three weeks post inoculation as shown in Fig. 3k.

### Selection of transformed hairy roots by GFP

**Fig. 4:**
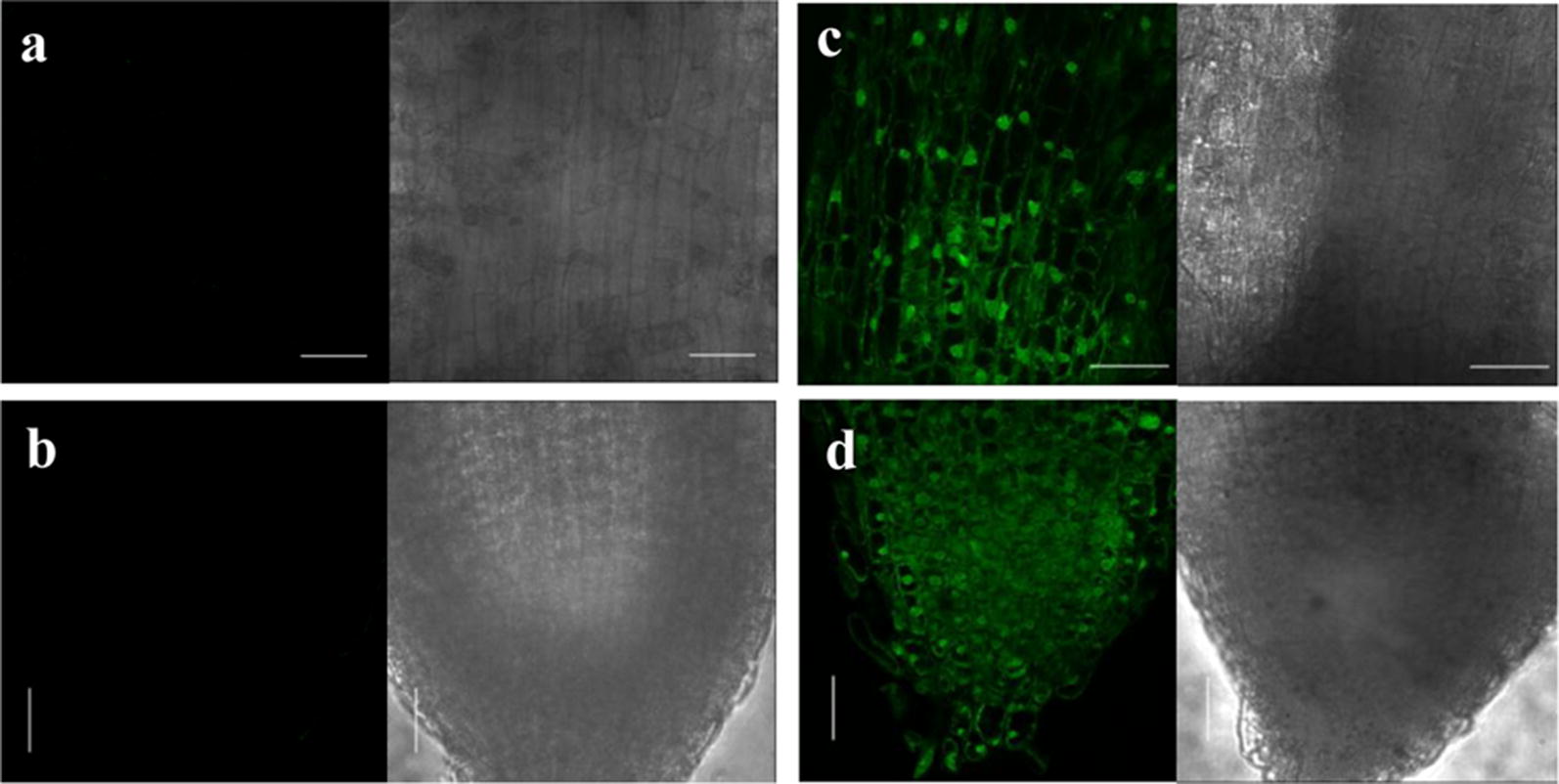
Green fluorescent protein (GFP) expression in transgenic chickpea (Annigeri) roots. GFP-derived fluorescence, bright field and merged image detected by laser scanning confocal microscope in a wild-type untransformed root (**a**, **b**) and transformed hairy root (**c**, **d**). Scale bar represents 100 µm

**Fig. 5:**
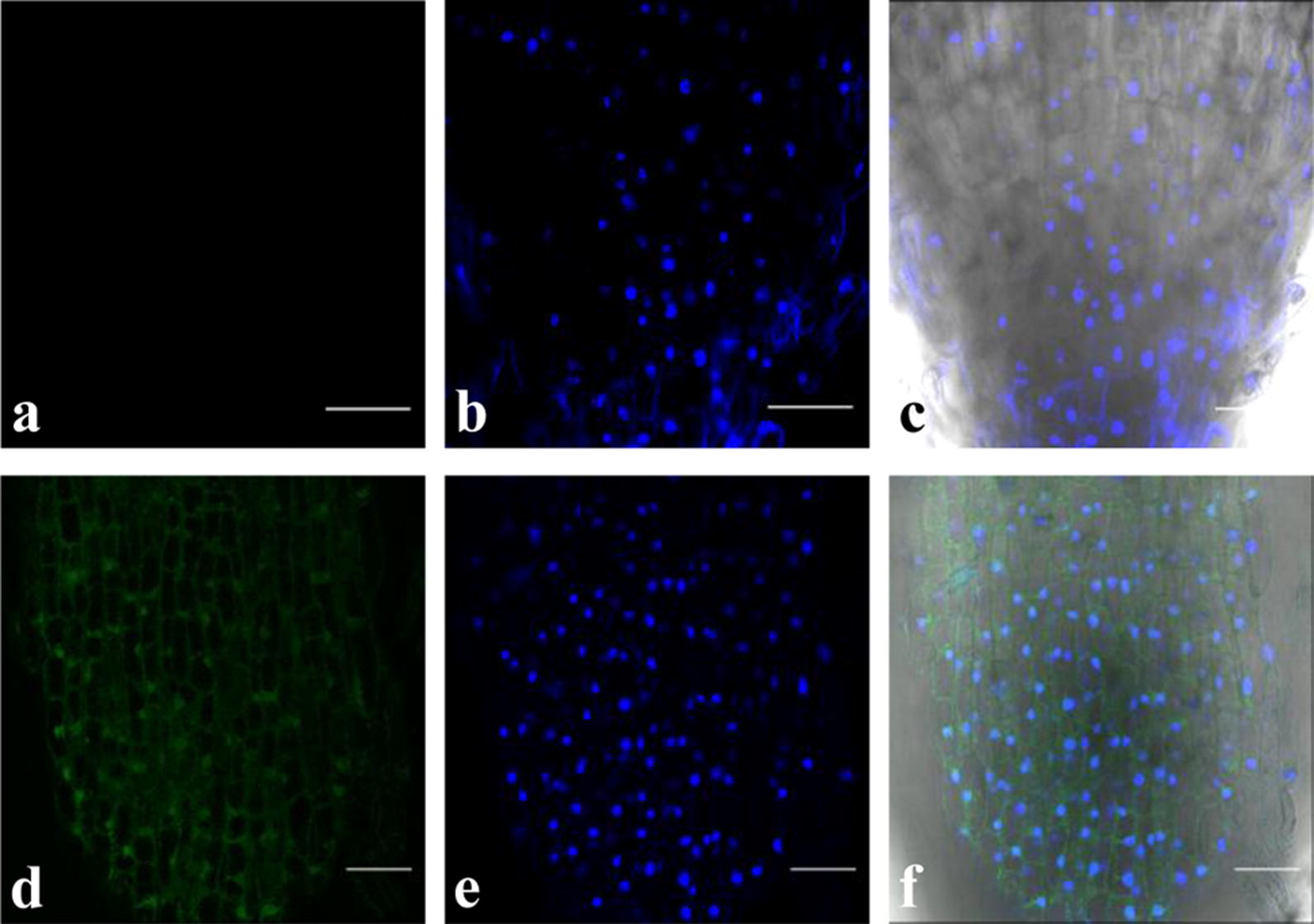
Characterization of transformed roots in chickpea (Annigeri). **a** No GFP expression in root tip, **b** DAPI staining exhibiting intact nuclei, **c** merged image with bright field in untransformed wild-type chickpea roots. **d** Visualization of GFP expression in root tip, **e** DAPI staining exhibiting intact nuclei, **f** merged image with bright field in transformed chickpea roots. Scale bar represents 100 µm

Green fluorescent protein (GFP) expression was visualized in the roots transformed with *A. rhizogenes* K599 harbouring the binary vector pCAMBIA1302 which contains GFP ORF under the control of CaMV35S promoter. A strong fluorescent signal was detected in the transformed root as shown in Fig. 4 and Additional file 2: Fig. S2. DAPI staining of transformed roots confirmed the integrity of nuclei after transformation (Fig. 5). Images of transformed roots along with untransformed as well as mock inoculated roots stained with DAPI are provided in Additional file 2: Fig. S3. At least three replicates were used to improve the statistical significance.

### Determination of the transformation efficiency in different chickpea cultivars

The transformation efficiency using *A. rhizogenes* strain K599 in different cultivars of chickpea was calculated based on both GFP expression as well as PCR confirmation to determine which chickpea cultivar is most amenable for root transformation and relevance of this protocol for future interventions during the establishment of genotype-independent transformation system. Seven chickpea cultivars namely, Annigeri, C-235, CPS 1, JG-62, K850, Vijay and WR-315 were tested in this study. Visualization of the expression of GFP in different cultivars with untransformed as well as mock inoculated roots is given in Additional file 2: Fig. S4. Integration of transgene was confirmed by PCR analysis of genomic DNA extracted from transformed and untransformed wild-type roots. Size of the amplification product using gene specific primers was expected (642 bp) and identical to K599 strain transformed with binary vector used as a positive control (Additional file 2: Fig. S5). However, no amplification was detected in wild-type roots. Also, the amplification of *virD* was not observed in any of the transformed roots suggesting the absence of *Agrobacterium* contamination. Based on the mean transformation efficiency, the individual chickpea cultivars were categorized into highest (above 60%), high (between 30%-60%) and low (below 30%) transformation amenable cultivars. We observed that Annigeri and JG-62 showed highest efficiency followed by K850, CPS 1, Vijay and C-235 showing high efficiency. Lowest transformation efficiency was observed in the cultivar WR-315. The results were reproduced from three independent experiments and the transformation efficiency of all chickpea cultivars is given in Table 3. To date, few studies have established *A. rhizogenes*-mediated root transformation in chickpea, however the efficiencies reported in these studies are very low (3–13%). In contrast, various parameters including co-cultivation temperature and duration have been optimized in the present study to achieve high transformation efficiency (23-61%) (Additional file 1: Table S1).

**Table 3:** Transformation efficiency of different chickpea cultivars. Seven chickpea cultivars inoculated with *A. rhizogenes* K599 strain harbouring binary vector pCAMBIA 1302

Genotype	Total number of explants inoculated	Explants with antibiotic selection positive roots (%)	Explants with GFP positive roots (%)^**^
Annigeri	50	73.50 (± 1.32)^a*^	61.62 (± 4.58)^a^
C-235	45	41.51 (± 2.11)^d^	35.58 (± 2.67)^c^
CPS 1	40	54.25 (± 3.14)^c^	41.65 (± 1.48)^b^
JG-62	49	72.14 (± 3.81)^a^	60.50 (± 2.63)^a^
K850	45	54.33 (± 0.99)^c^	40.57 (± 0.66)^b^
Vijay	40	63.33 (± 1.44)^b^	44.16 (± 1.44)^b^
WR-315	44	29.59 (± 2.94)^e^	23.51 (± 1.81)^d^

Each value represents the mean of three independent experiments with standard deviation (SD). Approximately 40–50 seedlings were examined for each individual experiment

^∗^Values in the same column followed by different letters showed significant difference among different transformation methods (Fisher's LSD test, *P *< 0.05)

^**^Corresponds to the frequency of seedlings that give rise to both GFP fluorescence and PCR positive roots

### Expression of *AtTT2* in chickpea hairy roots and quantification of soluble PAs

**Fig. 6:**
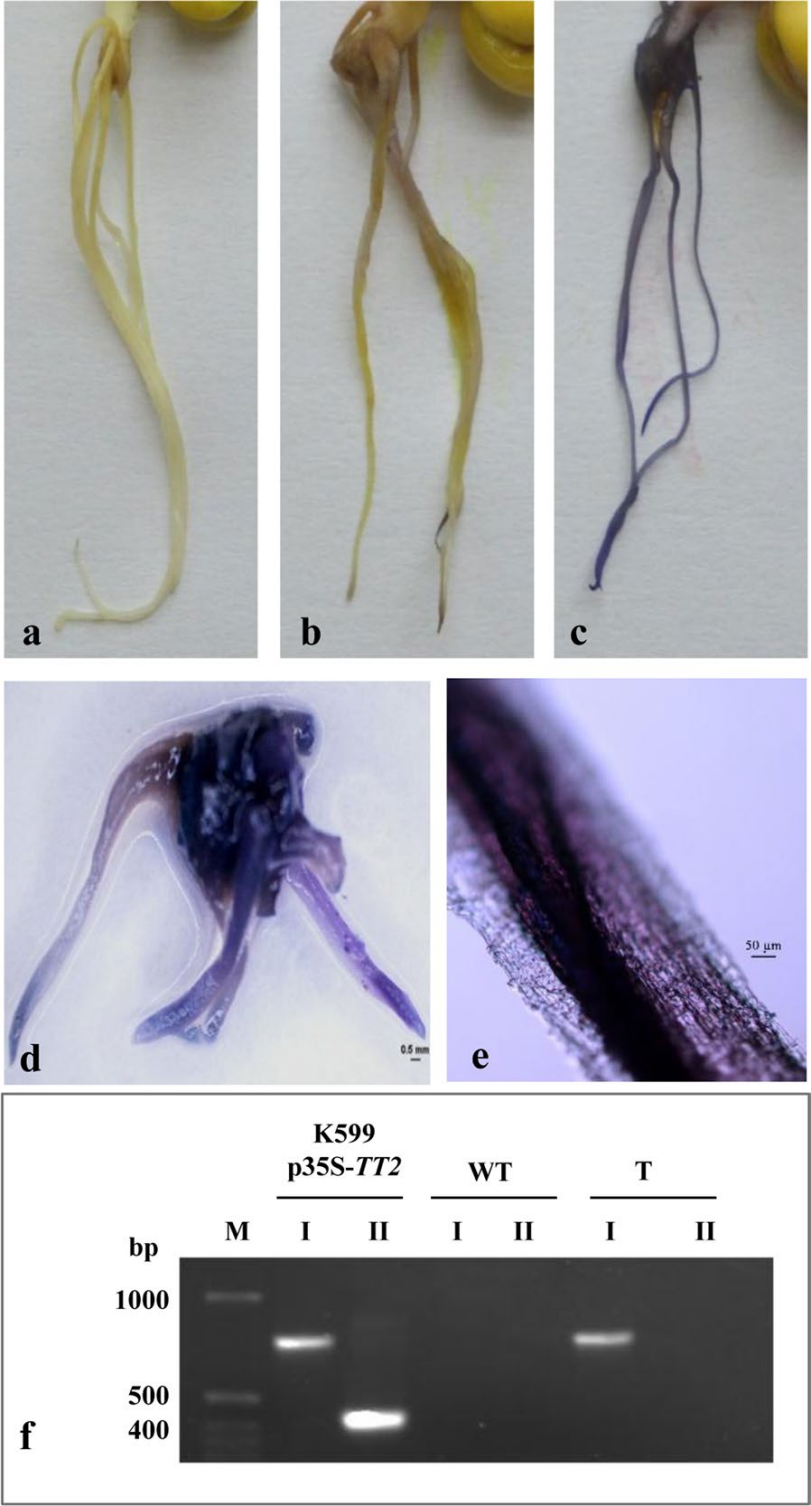
PA accumulation in transgenic roots of chickpea cultivar JG-62 expressing AtTT2:GFP. **a** Wild-type, **b** mock transformed and, **c**–**e** *AtTT2*-transformed roots after staining with DMACA reagent, **f** PCR amplification of *TT2* (I) and *virD* (II) genes in *A. rhizogens* K599-p35S-*TT2,* untransformed wild-type (WT) and transgenic roots (T) and lane M is a 1-kb ladder (Invitrogen). Scale bars: for **d** 0.5 mm and for **e** 50 µm

**Fig. 7:**
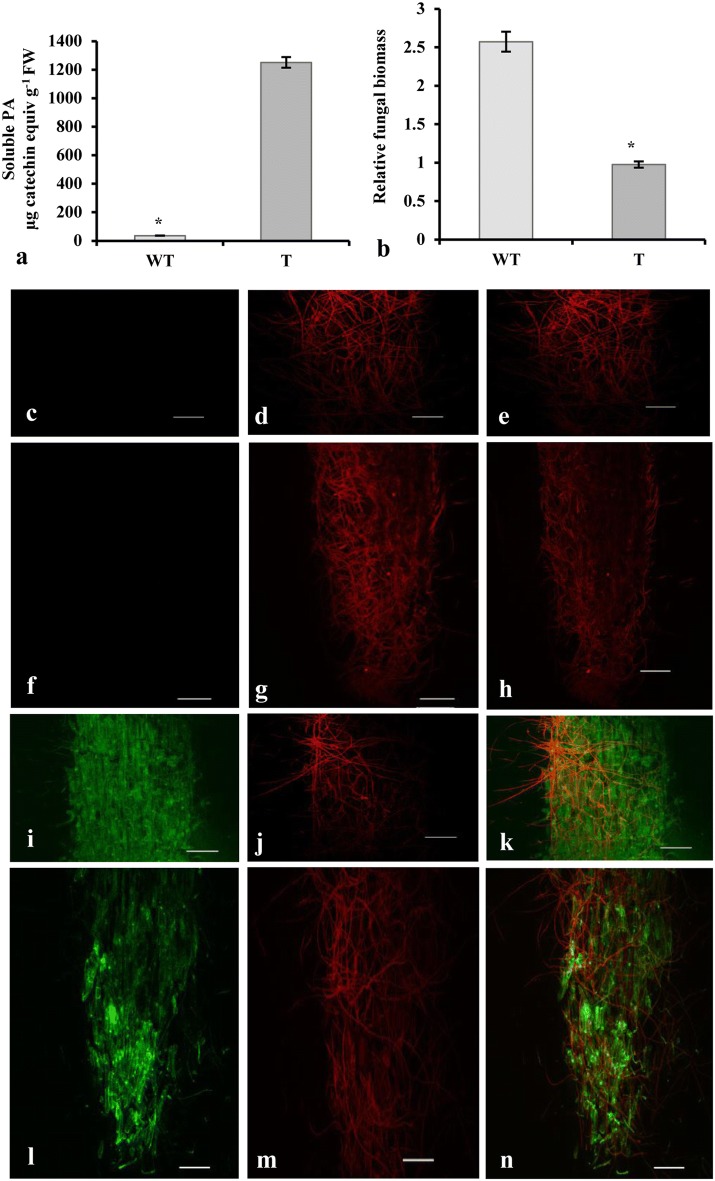
PAs accumulation leads to decreased fungal biomass in transformed chickpea (JG-62) roots. **a** Levels of proanthocyanidins (PAs) in untransformed wild-type (WT) and transgenic roots (T), **b** relative fungal biomass estimation in untransformed wild-type (WT) and transgenic roots (T). Each value represents the mean of three independent experiments with standard error (SE). Approximately 3-5 replicates were examined for each individual experiment. Statistically significant differences are indicated by an *asterisk* at *P *< 0.05 (Fisher’s LSD test). After 5 DPI, untransformed roots showing **c**, **f** no GFP fluorescence, **d**, **g** high fungal colonization, **e**, **h** merged image of root elongation and tip zones, respectively. *AtTT2*:GFP transformed roots showing **i**, **l** GFP fluorescence, **j**, **m** less fungal colonization, **k**, **n** merged image of root elongation and tip zones, respectively. Scale bars: 100 µm

Hairy roots can serve as an important tool for reverse genetics studies and gene expression analyses [22, 55, 56]. We further extended our study to validate the applicability of this method by expressing *Arabidopsis TRANSPARENT TESTA 2* (*AtTT2*) gene in chickpea hairy roots. *AtTT2* is known to be involved in proanthocyanidins (PAs) biosynthesis [47]. PAs are oligomeric and polymeric end products of flavonoid biosynthetic pathway that play important role in plant defence, such as protection against UV light damage, mechanical wounding, insect infestation and fungal infection [57–62]. Precisely, accumulation of proanthocyanidins in barley and poplar has been shown to potentially inhibit the growth of fungal pathogens, *Fusarium* species and *Dothiorella gregaria,* respectively [63, 64]. In our study, the transgenic roots expressing *TT2* showed intense blue colour after staining with DMACA, indicating the presence of PA polymers in the hairy roots (Fig. 6). Staining of whole chickpea plant expressing *TT2* exhibited the development of strong blue colour in the transgenic roots without any visible chimera. However, the colour was not observed in the wild-type roots. The staining of wild-type, mock inoculated and hairy root is shown in Additional file 2: Fig. S6. Also, the PCR analysis of roots expressing *AtTT2* along with the positive and negative control is provided in Fig. 6f. Further, quantification of soluble PAs was performed, which confirmed the concomitant accumulation of high soluble PAs (approximately 1.2 mg catechin equivalents per g fresh weight) in transgenic roots, as compared to wild-type roots (Fig. 7a).

### Inoculation of chickpea hairy root by *Fusarium oxysporum*

To explore the applicability of chickpea hairy roots in studying stress response, we conducted infection assay using fungal pathogen, *Fusarium oxysporum* f. sp. *ciceri*. Previously, we have shown that this fungal strain infects chickpea [8]. Given that the accumulation of PAs led to enhanced resistance in host plants, we utilized hairy roots expressing *TT2* for infection at 5 DAI (days after inoculation) and found that the fungal biomass was significantly reduced in transgenic roots, as compared to wild-type (Fig. 7b). Also, the colonization in transformed roots was visualized by staining with wheat germ agglutinin-TMR (WGA-TMR) for fungal chitin. In consistence with the quantitative data, fungal colonization was limited in the roots expressing *TT2* (Fig. 7c–n).

Overall, the current method can be implemented for discerning root-rhizosphere interaction in chickpea. The contrasting differences in the tolerance/susceptibility of different cultivars further highlight the requirement of an efficient transformation protocol for a number of genotypes, which can be used further to understand the molecular mechanism underlying differential stress response in plants induced by pathogen and/or other environmental factors, such as dehydration, heat and salinity.

## Conclusion

*Agrobacterium rhizogenes*-mediated root transformation has largely been used to analyze gene functions in various crops. Among legumes, it was first demonstrated in *Lotus corniculantus* which was further utilized to study root nodule development [65, 66]. Subsequently, the technique has been used for investigating root-microbe interactions, over-expression and knock-down of key genes for functional characterization [43]. In legumes, this method has a wide applicability as several pathogens invade the plants from roots and legumes are known for their symbiotic association with rhizosphere microbes. Among legumes, research on chickpea is gaining momentum due to its agronomic importance and being a major source of dietary protein. However, the recalcitrant nature of this crop has always been a limiting factor for genetic transformation using *A. tumefaciens*. Therefore, it is very important to develop an efficient transformation method to analyze function of biomarkers identified through various condition-dependent transcriptome and proteome studies. Here, we demonstrate an efficient, high-throughput and genotype-independent method of root transformation in chickpea using *A. rhizogenes.* The protocol described in our study provides an opportunity to transform chickpea roots in less time with high competence. This method is faster, more efficient and reproducible than the conventional transformation methods using *A. tumefaciens*. Use of different chickpea cultivars shows the efficacy of the method in a genotype-independent manner that makes it more amenable for large-scale screening of biomarkers. Further, validation using *AtTT2* gene confirmed the applicability of our method. Besides, interestingly enough, overexpression of *AtTT2* enhanced the level of PAs in hairy roots, which might decrease the colonization of fungal pathogen, *F. oxysporum.* Previously, accumulation of PAs had been shown to potentially inhibit the growth of *Fusarium* species in barley [63]. Very recently, overexpression of *AtTT2* like gene *MYB115* has been shown to enhance fungal resistance in poplar [64]. Altogether, this protocol offers an opportunity to functionally characterize genes involved in root developmental processes, plant-pathogen and plant–microbe interaction as well as the interaction of root with rhizosphere and abiotic stress response.

## Methods

### Chemicals

LB agar (Invitrogen Cat. # 22700025), LB broth (Invitrogen Cat. # 127800520), Acetosyringone (SIGMA Cat. # D134406), Potato dextrose broth (HIMEDIA Cat. # M403), MS salt (SIGMA Cat. # M5524), sucrose (SIGMA Cat. # S0389), agar (SIGMA Cat. # A4550), Cefotaxime (SIGMA Cat. # 22128), Hygromycin (SIGMA Cat. # H9773), Kanamycin (SIGMA Cat. # K4378), 4-dimethylaminocinnamaldehyde (DMACA) (SIGMA Cat. # D4506), catechin (SIGMA Cat. # C1251). All other chemicals were purchased from SIGMA-Aldrich (St. Louis, MO).

### *Agrobacterium rhizogenes* strain and binary vector

*Agrobacterium rhizogenes* strain K599 also known as NCPPB2659 was obtained from National Collection of Plant Pathogenic Bacteria Central Science Laboratory, Sand Hutton, York YO 41 ILZ England (http://www.ncppb.com). Binary vector pCAMBIA1302 (CAMBIA) which contains GFP ORF under the control of CaMV35S promoter was introduced into *A. rhizogenes* strains by electroporation. *Agrobacterium* strain harboring the binary vector were streaked directly from glycerol stock onto LB agar plates supplemented with kanamycin (50 mg L^−1^) and incubated for 2 days at 25 °C. A single colony was grown in LB broth medium containing kanamycin (50 mg L^−1^) and incubated at 25 °C at 180 rpm. Secondary culture was inoculated into 50 mL LB containing kanamycin (50 mg L^−1^) from 0.1% of the overnight grown culture. 50 µL of 100 mM acetosyringone was added after OD reaches to 0.6 and incubated at 25 °C for 5 h for inducing virulence. The culture was resuspended in sterile distilled water containing 100 µM acetosyringone.

### Plant material and growth conditions

Chickpea (*Cicer arietinum* L.) seeds used in this study were obtained from ICRISAT, Hyderabad and multiplied at the experimental fields of NIPGR, New Delhi. Seeds were sterilized with 4% sodium hypochlorite for 15 min followed by washing with autoclaved water for 7–8 times and soaked overnight in the dark. Seeds were placed on MS salt mixture media (SIGMA) containing 0.6% agar (SIGMA) and incubated for 5 days in dark in the growth chamber. The temperature and humidity in growth room were maintained at 25 ± 2 °C and 50 ± 5% relative humidity under 16 h photoperiod (60 μM m^−2^ s^−1^).

### Explant preparation and transformation

Five days old healthy seedlings were used for transformation by cutting the radical near the collar region using a sterile scalpel. Radicals were immersed in the suspension of *A. rhizogenes* strain K599 harboring control plasmid or plasmid containing the gene of interest for 35 min at room temperature. Seedlings were placed on the Whatman #5 filter paper for 5 s and then transferred onto MS agar medium. Vials were incubated in dark for 4 days for co-cultivation at 22 °C and 70% humidity. Co-cultivation conditions were optimized by assessing the root transformation efficiencies at different temperatures (20, 22, 24, 26, 28 and 30 °C) and co-cultivation durations (0.5, 1, 2, 3, 4 and 5 days). After co-cultivation, seedlings were transferred to fresh vials containing cefotaxime (250 mg L^−1^), hygromycin (20 mg L^−1^) and incubated at 25 ± 2 °C with a 16 h photoperiod (60 μM m^−2^ s^−1^). In parallel, control plants were immersed in sterile water and transferred onto the MS medium with or without antibiotic. For GFP visualization and stress treatment, transformed roots as well as the untransformed plantlets were further transferred to fresh MS medium without antibiotic or into pots containing a mixture of agropeat (Prakruthi Agro Tech, India) and vermiculite (1:1).

Alternatively, transformation by injection method was performed as described by Estrada-Navarrete et al. [40]. Briefly, five days old plantlets with unfolded cotyledons were pricked and inoculated by direct injection into the cotyledonary nodes using a sterile syringe. Approximately, 5–10 µL of the inoculum was injected into the wound for three times at different positions around the node. After inoculation, plants were transferred to the pots, kept in tray covered with a plastic lid and incubated in a growth chamber at 25 °C (16 h/8 h light and dark photoperiod).

### Screening of GFP positive roots

Ten days after inoculation or seven days after transferring the plantlets onto selection medium, roots were collected from transformed as well as control plants. Root sections were fixed and embedded following Ferguson and Reid [67] with modifications. Briefly, the samples were fixed in 4% (v/v) formaldehyde for 2 h followed by dehydration in graduated series of ethanol for 3 h. Further, the sections were treated with xylene for 5 h and embedded in paraffin (MERCK). Sections were prepared with a Rotary microtome (Leica). The surface area of each root was scanned for GFP visualization using confocal microscope (Leica SP2 LCM). Fluorescence signals were observed using 488 nm excitation and 520 nm emission filters.

### Histochemical staining of *AtTT2*-transformed roots and visualization

Histochemical analysis of PA accumulation in chickpea roots transformed with *TT2* was performed as described by Pang et al. [68]. In brief, roots were immersed in 0.5% (w/v) DMACA in ethanol and 6 M HCl (1:1, v/v) for 3 h. Images of stained roots were recorded using AZ100 stereozoom microscope (Nikon) and Eclipse 80i microscope (Nikon).

### DNA extraction and PCR analysis

DNA from transformed and wild-type roots was extracted using DNeasy Plant Mini Kit (QIAGEN). The following primers were used for the amplification of 642 bp and 438 bp fragments of *GFP* and *virD*, respectively (*GFP* forward 5′ GTAAACGGCCACAAGTTCAGCG 3′, *GFP* reverse 5′ TCGTCCATGCCGAGAGTGATCC 3′; *virD* forward 5′ ATGTCGCAAGGACGTAAGCCGA 3′, *virD* reverse 5′ GGAGTCTTTCAGCATGGAGCAA 3′). The PCR reaction mixture was as follows: 10 ng of plant genomic DNA, 2.5 μL of 10× PCR buffer, 1.5 μL of 25 mM MgCl_2_, 1.0 μL of 2.5 mM dNTP, 1 unit of Phusion High-Fidelity DNA polymerase (Thermo Scientific), 1 μL of 10 pM forward and reverse primers in a final volume of 25 μL. PCR was carried out using the following cycle conditions: 98 °C for 30 s 1 cycle, 98 °C for 30 s, 60 °C for 30 s (for GFP) and 56 °C for 30 s (for virD), 72 °C for 45 s 30 cycles and a final extension at 72 °C for 10 min. Amplified products were electrophoresed on 1.5% agarose gel containing 0.5 mg L^−1^ ethidium bromide and visualised under UV light.

### Proanthocyanidins (PAs) quantification

Soluble PAs extraction was performed as described by Pang et al. [68]. Briefly, roots were ground in liquid nitrogen and 1 g of tissue was extracted with 5 mL of extraction solution (70% acetone, 0.5% acetic acid) followed by vortexing and sonication at 30 °C for 30 min. Samples were centrifuged at 2500 g for 10 min and residues were re-extracted twice as above. Supernatants from each extraction were pooled and extracted with 30 mL of chloroform. Aqueous supernatant was re-extracted twice with chloroform and three times with hexane. Samples were freeze dried and resuspended in the extraction solution to a final concentration of 3 g of original sample/mL. 2.5 µL aliquots of samples were mixed with 197.5 µL of DMACA reagent [0.5% (w/v) DMACA in methanol-3 N HCl (1:1)] in microwell plate. For blanks, the same samples were replaced with 2.5 µL of extraction solution. Catechin was used as a standard. For samples, blanks and standards absorbance was read at 640 nm on a POLARstar Omega (BMG LABTECH) plate reader within 15 min. Blanks were subtracted from samples and PA content was calculated as catechin equivalents.

### Fungal infection, visualization and biomass determination

For stress treatment, fungal strain *Fusarium oxysporum* f. sp. *ciceri race 1* was grown in 50 mL of potato dextrose broth (PDB) and incubated at 28 °C with 180 rpm for 7–8 days. *F. oxysporum* spores were filtered using sterile cheese cloth to remove the mycelium. Number of spores were counted using hemocytometer and spore suspension was diluted to the concentration of 1 × 10^6^ spores mL^−1^ with sterile distilled water. *C. arietinum* cultivar JG-62 transformed with *TT2* as well as wild-type were used for infection studies. Ten days old transformed roots were dipped into *F. oxysporum* spore suspension while the control plants were treated with water. Roots were harvested at three days post infection for microscopic analysis and fungal biomass determination.

Control and transformed roots infected with *F. oxysporum* were fixed at 5 DAI and stained with Wheat Germ Agglutinin, Tetramethylrhodamine Conjugate (Thermo Scientific, India), as described by Deshmukh et al. [69]. Briefly, root segments were fixed at room temperature for 2 h in 4% paraformaldehyde with 2 mM MgCl_2_, 2 mM EGTA and 0.1% Tween 20 (w/v). Fixed root segments were washed with 1 × PBS (phosphate buffer saline) and transferred into enzyme solution containing 10 mg mL^−1^ driselase, 10 mg mL^−1^ chitinase, 10 mg mL^−1^ proteinase K and 1 mg mL^−1^ BSA for 15 min at RT. After rinsing with PBS, root segments were treated with 0.5% Triton X-100, rinsed twice with PBS and used for subsequent staining. Screening of stained roots was done using a Leica SP2 LCM confocal microscope. Fluorescence signals were observed using 555 nm excitation and 580 nm emission filters.

The levels of *F. oxysporum* and chickpea DNA were determined using qRT-PCR with pathogen specific primers for *F. oxysporum glyceraldehyde 3-phosphate dehydrogenase (GPD)* forward 5′AAGGGTGCTTCTTACGACCA 3′, reverse 5′ ATCGGAGGAGACAACATCGT 3′ and chickpea *18s rRNA* forward 5′ CTCGGCCCAACTCCGGTTCG 3′, reverse 5′ CGCACGAAAACCGTCTCCGGT 3′. Relative fungal biomass was calculated by normalizing the Ct value of *F. oxysporum* *GPD* to chickpea *18s rRNA*.

### Statistical analysis

The data was compared by ANOVA followed by a comparison of means using Fisher’s LSD test. Values followed by different letters are significantly different at *P *< 0.05.

## Additional files

**Additional file 1.** **Table S1**. Comparison of method and transformation efficiency with previous studies.

**Additional file 2.** **Fig. S1**. Wild-type and transformed roots of chickpea cultivar Annigeri grown in selection medium. **Fig. S2**. Green fluorescent protein (GFP) visualization by confocal microscopy in transformed chickpea (cultivar Annigeri) roots. **Fig. S3**. Characterization of transformed roots in chickpea cultivar Annigeri. **Fig. S4**. Green fluorescent protein (GFP) expression in different chickpea cultivars. **Fig. S5**. PCR analysis of transgenic chickpea roots expressing GFP. **Fig. S6**. Characterization of roots of chickpea cultivar JG-62 expressing AtTT2:GFP.

## Abbreviations

EST: expressed sequence tags
Ti plasmid: tumor inducing plasmid
Ri plasmid: root inducing plasmid
rol: root locus
GFP: green fluorescent protein
WGA: wheat germ agglutinin
TMR: tetramethylrhodamine
