# Supplementary material for: Genotype-independent Agrobacterium rhizogenes-mediated root transformation of chickpea: a rapid and efficient method for reverse genetics studies
Source: Plant Methods. 2018 Jul 6;14:55. doi: 10.1186/s13007-018-0315-6 (PMC6034309; doi:10.1186/s13007-018-0315-6)
Supplement: Supplementary file 1 — Additional file 1. Table S1. Comparison of method and transformation efficiency with previous studies. [file 13007_2018_315_MOESM1_ESM.pdf]

**Table S1 Comparison of method and transformation efficiency with previous studies.** Comparison of genotype, explant, co-cultivation duration, co-cultivation temperature used in the current study and the transformation efficiencies obtained from this method with the previous studies.

| | Genotype | Explant | Cocultivation duration (days) | Cocultivation temperature ($^0$C) | Transformation efficiency (%) | Reference |
|---|---|---|---|---|---|---|
| Previous study | Red chickpea | Seedling | 2 | Not specified | 3.17 | Bajrovic., et al. (1997) |
| Previous study | Gökçe | Seedling | 2 | 24 | 17.5 | Khawar and Ozcan, (2004) |
| | Akçin 91 | Seedling | 2 | 24 | 13.5 | |
| | Izmır 92 | Seedling | 2 | 24 | 0 | |
| Current study | Annigiri | Seedling | 4 | 22 | 61.62 | |
| | JG-62 | Seedling | 4 | 22 | 60.50 | |
| | Vijay | Seedling | 4 | 22 | 44.16 | |
| | CPS-1 | Seedling | 4 | 22 | 41.65 | |
| | C235 | Seedling | 4 | 22 | 35.58 | |
| | K850 | Seedling | 4 | 22 | 40.57 | |
| | WR-315 | Seedling | 4 | 22 | 23.51 | |
