# Supplementary material for: Genotype-independent Agrobacterium rhizogenes-mediated root transformation of chickpea: a rapid and efficient method for reverse genetics studies
Source: Plant Methods. 2018 Jul 6;14:55. doi: 10.1186/s13007-018-0315-6 (PMC6034309; doi:10.1186/s13007-018-0315-6)
Supplement: Supplementary file 2 — Additional file 2. Fig. S1. Wild-type and transformed roots of chickpea cultivar Annigeri grown in selection medium. Fig. S2. Green fluorescent protein (GFP) visualization by confocal microscopy in transformed chickpea (cultivar Annigeri) roots. Fig. S3. Characterization of transformed roots in chickpea cultivar Annigeri. Fig. S4. Green fluorescent protein (GFP) expression in different chickpea cultivars. Fig. S5. PCR analysis of transgenic chickpea roots expressing GFP. Fig. S6. Characterization of roots of chickpea cultivar JG-62 expressing AtTT2:GFP. [file 13007_2018_315_MOESM2_ESM.pdf]

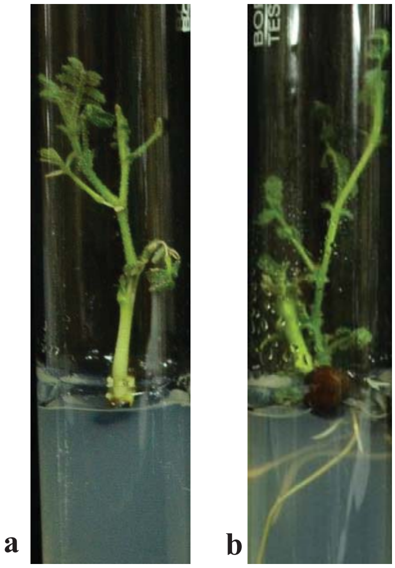

Fig. S1 Wild-type (**a**) and transformed roots (**b**) of chickpea cultivar Annigeri grown in selection medium.

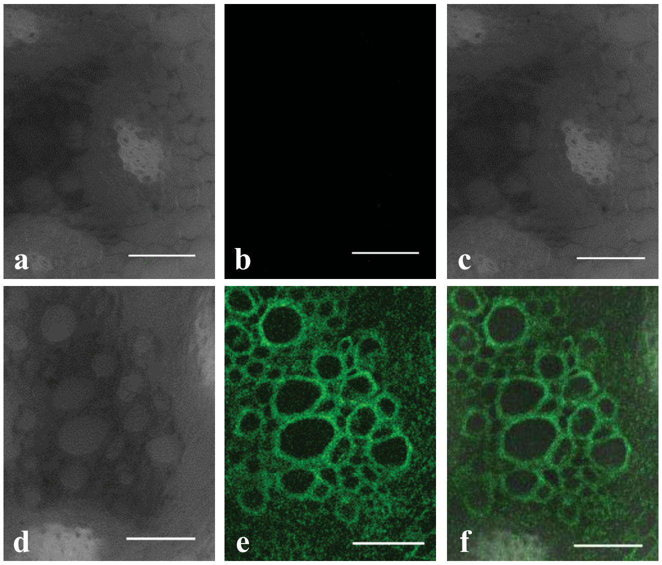

Fig. S2 Green fluorescent protein (GFP) visualization by confocal microscopy in transformed chickpea (cultivar Annigeri) roots. Cross sections observed under bright field, GFP fluorescence and overlay of bright-field and fluorescence image of (**a-c**) the untransformed wild-type and (**d-f**) transformed roots. Scale bar: 100 μm.

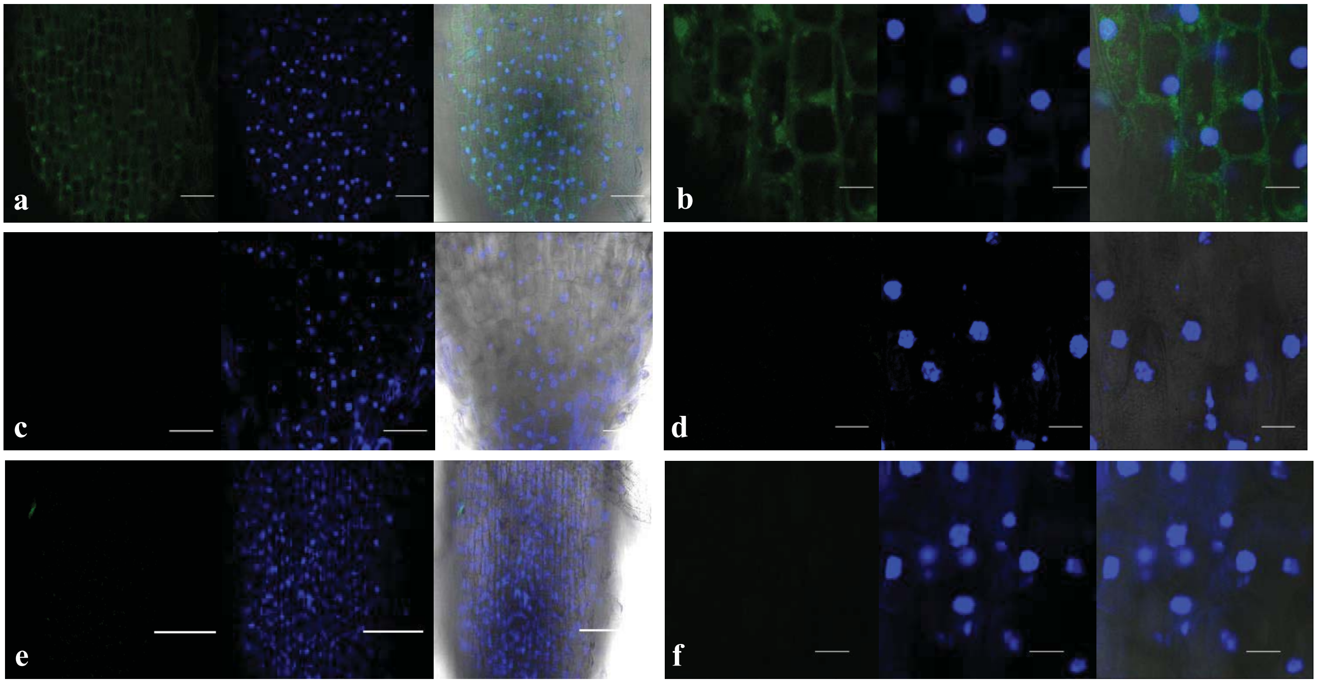

Fig. S3 Characterization of transformed roots in chickpea cultivar Annigeri. Visualization of GFP expression in root tip, DAPI stating exhibiting intact nuclei, and merged image with bright field of (a, **b**) in transformed chickpea roots; (**c, d**) untransformed wild-type, and (**e, f**) mock inoculated roots. Scale bars: (**a**, **c**, **e**) 100 µm and (**b, d**,**f**) 40 µm.

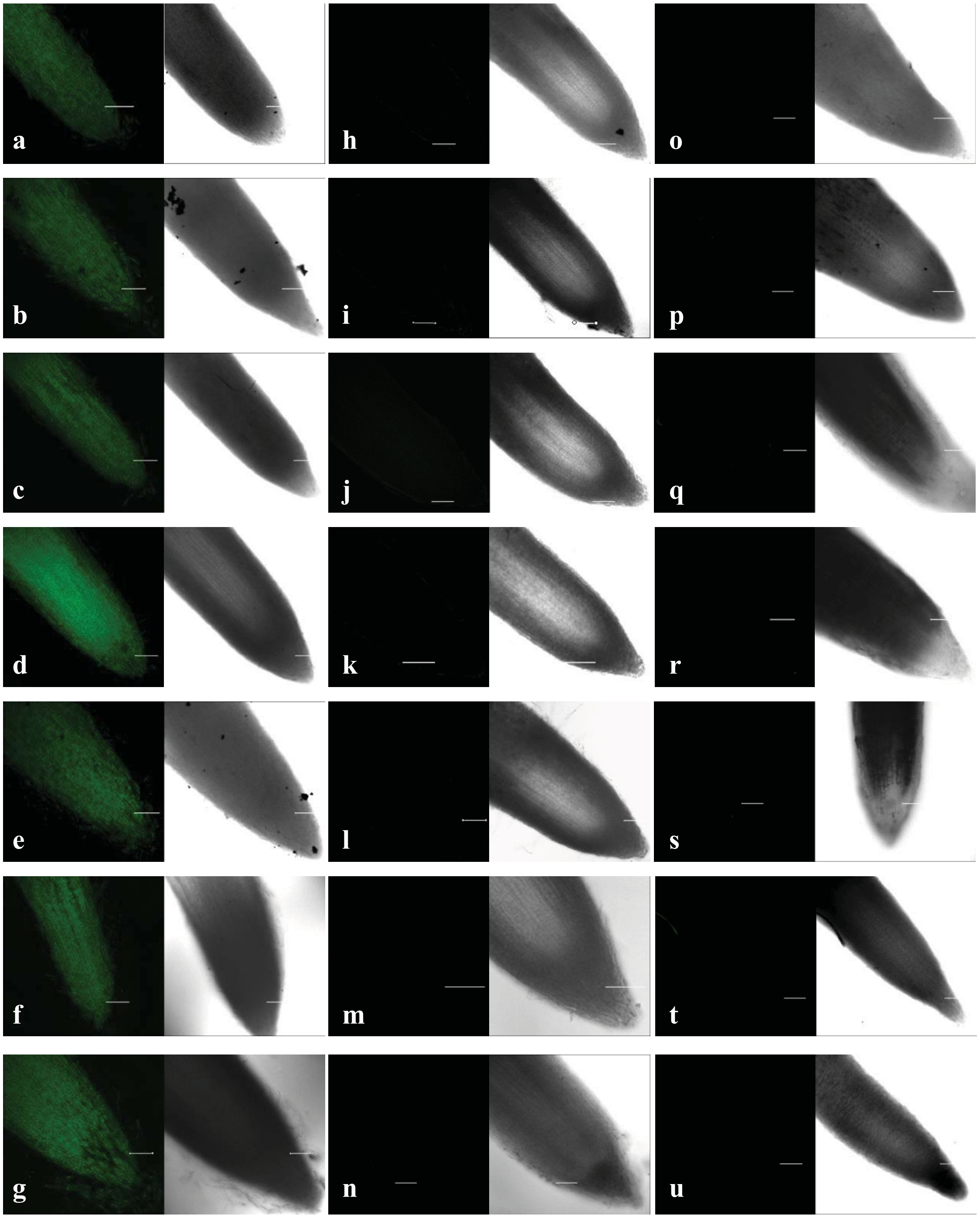

Fig. S4 Green fluorescent protein (GFP) expression in different chickpea cultivars. GFP-derived fluorescence as well as bright field image detected by laser scanning confocal microscope in a transformed hairy root, untransformed wild-type and mock-inoculated roots of chickpea cultivar (**a, h, o**) Annigeri, (**b, i, p**) JG-62, (**c, j, q**) Vijay, (**d, k, r**) C104, (**e, l, s**) K850, (**f, m, t**) C235, (**g, n, u**) WR-315, respectively. Scale bar represents 100 µm.

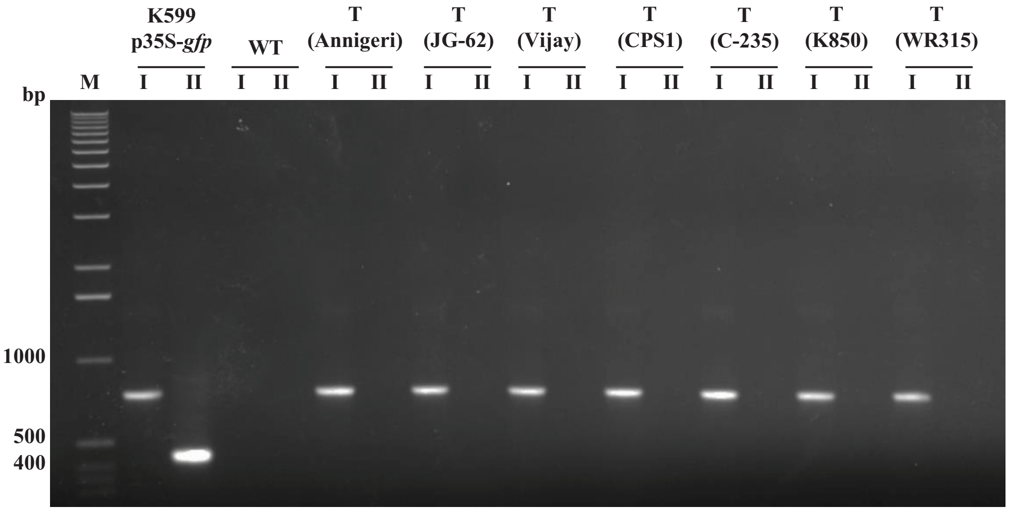

Fig. S5 PCR analysis of transgenic chickpea roots expressing GFP. PCR amplification of GFP (I) and virD (II) genes in *A. rhizogens* K599-p35S-*gfp,* untransformed wild-type (WT) and transgenic roots (T) of different chickpea cultivars. Lane M is a 1-kb ladder (Invitrogen).

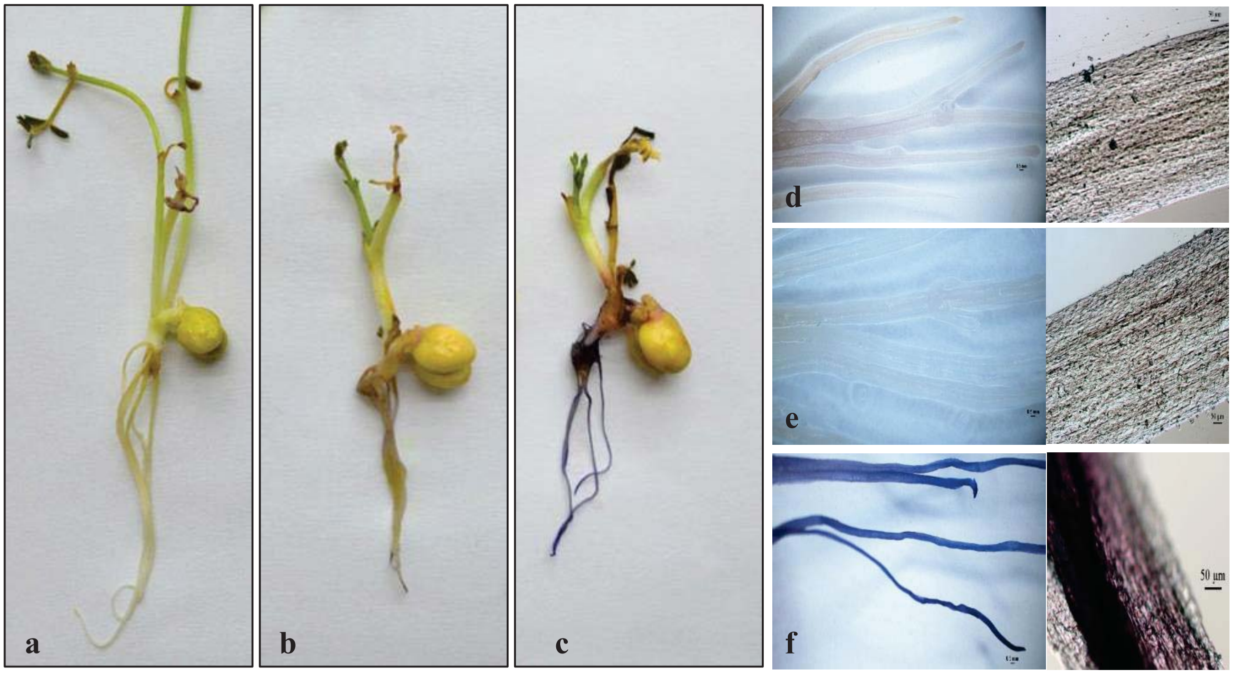

Fig. S6 Characterization of roots of chickpea cultivar JG-62 expressing AtTT2:GFP. DMACA staining of (**a, d**) wild-type, (**b, e**) mock inoculated and (**c, f**) transgenic roots expressing *AtTT2*. Scale bars: (**d, e, f**) le`ft panel 0.5 mm and right panel 50 µm.
